# Outcome differences after orthodontic camouflage treatment in hypo- and hyperdivergent patients – A retrospective cephalometric investigation

**DOI:** 10.1007/s00784-023-05321-7

**Published:** 2023-11-13

**Authors:** Jan Hourfar, Gero Stefan Michael Kinzinger, Linda Frye, Jörg Alexander Lisson

**Affiliations:** Department of Orthodontics, Saarland University, 66424 Homburg, Saar Germany

**Keywords:** Extraction, Hypodivergent, Hyperdivergent, Camouflage treatment, Bite opening

## Abstract

**Objectives:**

To compare differences in outcome in skeletal and dental parameters in hypo- and hyperdivergent Class II patients after extraction of upper first premolars and comprehensive orthodontic treatment.

**Materials and methods:**

37 Class-II-patients with dental camouflage treatment were divided into a hypo- (*n* = 18) or a hyperdivergent (*n* = 19) group depending on the mandibular plane angle (hypo: < 34° or hyper: ≥ 34°). Lateral cephalograms were available before (T1) and after (T2) treatment and were analyzed with customized measurements. Data from a growth survey served as a control and were used to calculate the actual treatment effect. Data were analyzed by one-sample Student’s t-tests and independent Student’s t-tests. Statistical significance was set at *p* < 0.05.

**Results:**

The measurements showed similar changes in both groups. The effects were mainly dentoalveolar. Hypodivergent patients showed an almost equal increase in anterior and posterior facial height, while hyperdivergent patients only showed an increase in anterior facial height.

**Conclusions:**

In hyperdivergent patients, the anterior facial height increases despite camouflage treatment. This indicates a tendency towards bite opening and backward rotation of the mandible. Hypodivergent patients do not experience deepening of the bite.

**Clinical relevance:**

In hyperdivergent patients with upper first premolars extraction the anterior facial height increased differently than in hypodivergent patients. This should be considered if a bite opening is a possible contraindication to treatment.

## Introduction

Class-II is the most common malocclusion in Caucasian populations [1] and marked by a distobasal jaw relationship. Approximately 80% of the affected patients display a retrognathic mandible, while the remaining 20% exhibit maxillary prognathism [2].

Occlusal Class II correction is possible by causal (skeletal correction) and non-causal (dentoalveolar compensation/camouflage) treatment. Causal skeletal correction is possible through removable functional appliances (RFA), e.g. bionator, and through fixed functional appliances (FFA), which work without patient compliance and provide a permanent effect [3–5]. Various FFA are described and thoroughly investigated in the literature [6–9]. It is recognized that hyperdivergent patients can react unfavorably to FFA-treatment [2], e.g. with a bite opening.

Although there are only few studies on their effects in hypo- or hyperdivergent growth types [5, 10], hypodivergent facial growth is considered a relative contraindication to FFA treatment, requiring either a surgical approach or a dentoalveolar compensation.

If mandibular advancement is no option for hyperdivergent patients, dental camouflage (DC) treatment with extraction of upper premolars can be performed alternatively [11]. Premolars are probably most frequently extracted for orthodontic purposes, as they are conveniently located between the anterior and posterior segments [12]. After overjet correction by retraction of the anterior segment, the mandibular position remains unchanged. The skeletal class-II-discrepancy is thus merely concealed by the selective removal of permanent teeth [21]. Consequently, DC treatment is regarded as an alternative for patients with hyperdivergent facial growth, because this approach has been described»to close down the bite« [13]. However, there is hardly any literature on DC, especially not differentiating between hypo- and hyperdivergent facial growth.

The comparison of vertical effects after dental camouflage treatment in patients with hypo- and hyperdivergent facial growth types has not yet been done. According to the PICO scheme, this retrospective study investigated juvenile and/or adolescent patients with hypo- and hyperdivergent facial growth types and a skeletal Class II (P) with dental camouflage treatment (I) were compared to each other and to an untreated control (C) and were scrutinized for:

- selected skeletal and dentoalveolar changes (O) and
- possible side effects on occlusal plane, gonial angle and lower incisor inclination (O).

## Material and methods

### Patients

*N* = 37 patients received dental camouflage treatment by the same experienced orthodontist for a skeletal Class II malocclusion defined by an ANB ≥ 4° and a distal occlusion of at least ½ cusp width before treatment.

Further inclusion criteria were:

- complete permanent dentition (except for third molars),
- no unintended tooth loss during treatment,
- no previous orthodontic treatment.

The exclusion criteria were:

- craniofacial anomalies,
- loss or agenesis of permanent teeth (except for third molars),
- previous extraction therapy.

No age restrictions were applied. *N* = 42 patients were screened for eligibility. *N* = 37 patients were included after 5 patients dropped out due to various reasons. The examination was performed on each patient at T1 and T2, with T1 recorded prior to and T2 immediately after treatment completion.

Following a study by Rogers et al. [5], patients were divided into a hypo- or hyperdivergent group depending on their pretreatment mandibular plane angles < 34° (hypodivergent) or ≥ 34° (hyperdivergent).

The hypodivergent group included *n* = 18 patients (9 males, 9 females) with a pre-treatment age (T1) of 11.75 ± 2.21 years, and the hyperdivergent group included *n* = 19 patients (9 males, 10 females) with a pre-treatment age (T1) of 12.26 ± 3.85 years. The mean age difference at T1 was not significant (*p* = 0.727). A preliminary evaluation of the mandibular plane angle showed that there was a highly significant (*p* < 0.001) difference between the groups at T1.

The sample size was calculated based on a significance level of 0.05 and a power of 80% to detect a clinically meaningful difference of 2.0 (± 2.0 mm/degrees) [21]. The power analysis revealed that 17 patients were necessary for each group.

A control group was created from the growth study by Bhatia and Leighton [14] to ensure comparability with similar studies [15, 16]. Their data came from Caucasian subjects participating in a longitudinal study of facial growth at King’s College in London/UK.

Matching of control and study groups at T1 and T2 was based on chronological age instead of skeletal maturation stage, as described in another study [17]. The difference between T1 and T2 in the control group represented natural growth effects that were unaffected by orthodontic treatment. This difference was subtracted from the delta between T1 and T2 of the study group. The resulting value then represented the treatment effect, referred to as the “Net effect”.

### Treatment protocol

All patients received comprehensive fixed appliance treatment with an MBT 0.022’’ bracket system (Sprint®-Brackets (Forestadent, Pforzheim, Germany). After being informed about the treatment options for skeletal Class II malocclusions, the patients decided against FFA or surgical advancement of the mandible and preferred a dental camouflage approach. After initial levelling and alignment with NiTi archwires starting with 0.012’’ up to 0.016’’, maxillary first premolars were extracted [11]. Dental arches were then aligned with stainless steel archwires from 0.016’’ × 0.022’’ up to 0.017’’ × 0.025’’. Retraction of the anterior segment was performed using a 0.017’’ × 0.025’’ TMA asymmetrical "T" archwire according to Hilgers and Farzin-Nia [18] without additional anchorage.

### Lateral cephalograms

Cephalometric radiographs were available for all patients before (T1) and after (T2) treatment. They were recorded using an analogue X-ray machine (Orthophos®, Sirona, Bensheim, Germany) with standardized conditions regarding head posture and maximal intercuspation. All images included a scale for the enlargement factor calculation. The radiation data varied between 73 kV/15 mA and 77 kV/14 mA depending on patient height and weight, exposure time was always 9 s. The lateral cephalograms were digitized and analyzed using dedicated tracing software (fr-win®, version 7.0, Computer Konkret, Falkenstein, Germany) with an accuracy of two decimals on a certified image viewing system for radiographic diagnostics.

**Fig. 1:**
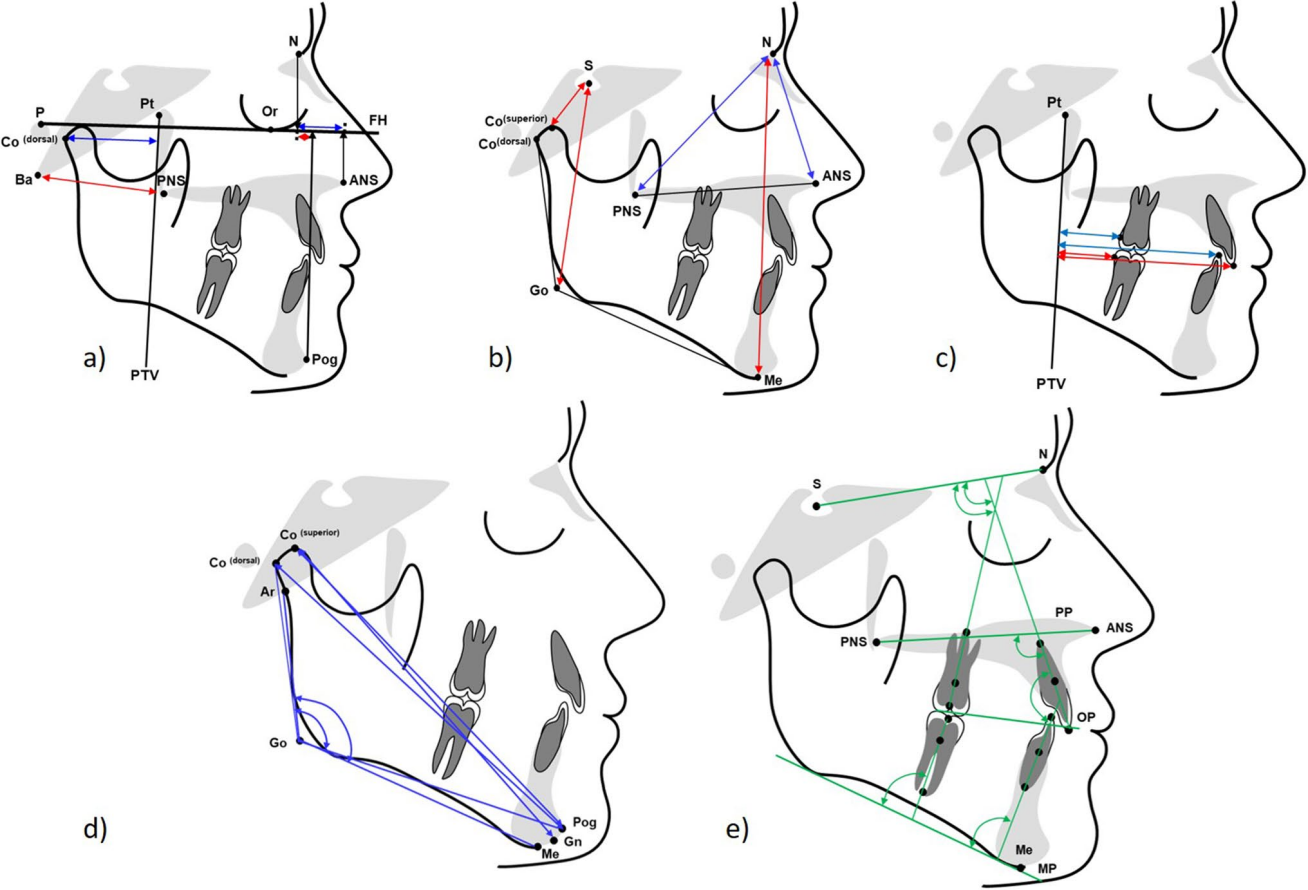
Skeletal and dental cephalometric measurements. **a**) horizontal linear: Co^(dorsal)−^PTV; Ba-PNS; N-ANS on FH; N-Pog on FH. **b**) vertical linear: S-Co^(superior)^; S-Go; N-Me; N-ANS; N-PNS. **c**) dentoalveolar linear: U1^(incisal)^-PTV; L1^(incisal)^-PTV; U6^(dorsal)^-PTV; L6^(dorsal)^-PTV. **d**) mandibular angular and linear: Co^(dorsal)^-Pog; Co^(superior)^-Gn; Ar-Go-Me; Co^(dorsal)^-Go-Pog (»modified gonial angle«);. **e**) dentoalveolar angular: SN/OP; U1/SN; U1/PP; L1/MP; U1/ L1 (interincisal angle); U6/SN; L6/MP

Hand-wrist x-rays were not routinely taken, respecting the ALARA [19]-principle. The control group data [14] also used chronological age rather than stages of skeletal maturity. The lateral cephalograms were analyzed according to Kinzinger et al. [16, 20] by a single blinded examiner to ensure comparability with other studies. Measurements are shown in Table 1 and Fig. 1. The analyses included maxillary and mandibular sagittal and vertical changes as well as sagittal dental changes.

**Table 1:** Cephalometric landmarks and measurements

Measurement	I. Skeletal and dental effects
Maxilla sagittal (mm)	
N-ANS on FH	anterior position of the maxillary base: linear distance between the junction of the frontal bone and nasal bone at the nasofrontal suture (Nasion (N)) and the most anterior point of the bony floor of the nose at the tip of the anterior nasal spine (ANS) projected onto the Francfort Horizontal (FH)
Ba-PNS	posterior position of the maxillary base: linear distance between the anterior margin of the foramen magnum (Basion (Ba)) and most posterior point of the bony floor of the nose at the tip of the posterior nasal spine (PNS)
Maxilla vertical (mm)	
N-ANS	linear distance between landmarks Nasion (N) and anterior nasal spine (ANS)
N-PNS	linear distance between landmarks Nasion (N) and posterior nasal spine (PNS)
Mandible sagittal (mm)	
N-Pog on FH	anterior position of the mandibular base: linear distance between landmark Nasion (N) and most anterior point of the bony chin (Pog) projected onto the Francfort Horizontal (FH)
Co^(dorsal)−^PTV	position of the dorsal condyle margin: linear distance between the most posterior point of the mandibular condyle (Co^(dorsal)^) and pterygoid vertical (PTV)
Mandible vertical (mm)	
S-Co^(superior)^	linear distance between the sella turcica’s midpoint (Sella, (S)) and condyle’s superior margin (Co^(superior)^)
S-Go	linear distance between landmark Sella (S) and intersection of the ramus tangent and corpus tangent (Go)
N-Me	linear distance between landmark Nasion (N) and most inferior point of the bony chin (Me)
Dental horizontal (mm)	
U1^(incisal)^-PTV	linear distance between the incisal tip of the upper central incisor (U1^(incisal)^) and PTV†
L1^(incisal)^-PTV	linear distance between the incisal tip of the lower central incisor (L1^(incisal)^) and PTV†
U6^(dorsal)^-PTV	linear distance between the most distal point of upper first molar’s tooth crown (U6^(dorsal)^) and PTV†
L6^(dorsal)^-PTV	linear distance between the most distal point of lower first molar’s tooth crown (L6^(dorsal)^) and PTV†
Overjet	distance between the incisal tips of the lower (L1^(incisal)^) and upper central incisors (U1^(incisal)^) measured along the occlusal plane (OP)
Overbite	distance between the tips of the lower (L1^(incisal)^) and upper central incisors (U1^(incisal)^) measured perpendicular to the occlusal plane (OP)
	II. Side effects on occlusal plane, gonial angle and lower incisor inclination
Mandible diagonal (mm)	
Co^(dorsal)^-Pog	linear distance between landmarks Co^(dorsal)^ and Pog
Co^(superior)^-Gn	linear distance between the most superior point of the mandibular condyle (Co^(superior)^) and most anterior, inferior point on the mandibular symphysis (Gnathion, (Gn))
Mandible angular (°)	
Ar-Go-Me	gonial angle: angle between intersection of the posterior border of the neck of the condyle with the cranial base (Ar) and landmarks gonion (Go), and menton (Me)
Co^(dorsal)^-Go-Pog	modified gonial angle: angle between the landmarks posterior condylar margin (Co^(dorsal)^), gonion (Go), and pogonion (Pog) landmarks
Cant of occlusal plane (°)	
SN/OP	angle between the anterior cranial base (SN) and the occlusal plane (OP)
Dental angular (°)	
U1 / SN	angle between the longitudinal axis of the upper central incisor (U1) and anterior cranial base (SN)
U1 / PP	angle between the longitudinal axis of the upper central incisor (U1) and palatal plane (PP)
L1 / MP	angle between the longitudinal axis of the lower central incisor (L1) and mandibular plane (MP)
U1/ L1	interincisal angle: angle formed by the intersection of the longitudinal axis of the upper central incisor (U1) with the longitudinal axis of the lower central incisor (L1)
U6 / SN	angle between the longitudinal axis of the upper first molar (U6) and anterior cranial base (SN)
L6 / MP	angle between the longitudinal axis of the lower first molar (L6) and mandibular plane (MP)

†Measurement perpendicularly onto PTV

### Statistical analysis

The analyses were performed by scrutinizing lateral cephalograms that were routinely obtained during orthodontic therapy. 25% of the lateral cephalograms were randomly selected and re-traced after one month by the same examiner. The intrarater reliability was confirmed by calculating the method error (ME) using the Dahlberg-formula (ME = √(∑d^2^/2n)) [22]. ME was < 1 for linear (0.78 mm) and angular (0.57°) measurements. Normal distribution of the data was confirmed by the Shapiro-Wilk test. Homogeneity of variance was tested using Levene’s method. One-sample Student’s t-tests were applied for intragroup comparisons, and independent Student’s t-tests for intergroup comparisons. Descriptive statistics mean (M) and standard deviation (SD) were recorded for each variable. Additionally, 95% confidence intervals (95% CI) were calculated. All statistical analyses were performed using SPSS® Version 28 for Windows® (IBM Corp., Armonk, NY, USA). Statistical significance was set at *p* < 0.05.

## Results

Treatment time was 3.72 ± 1.99 years in hypodivergent and 3.90 ± 1.72 years in hyperdivergent patients. No significant difference existed (*p* = 0.811). All cephalometric measurement results are shown in Tables 2, 3, 4, 5, 6 and 7.

**Table 2:** Skeletal and dental effects in all patients

Extraction—all patients						
Measurement	T1 (M ± SD) 95% CI (LB, UB)	T2 (M ± SD) 95% CI (LB, UB)	∆T2-T1 (M ± SD) 95% CI (LB, UB)	Control (M ± SD) 95% CI (LB, UB)	Net (M ± SD) 95% CI (LB, UB)	Net *p*-value (intra)
Maxilla sagittal (mm)						
N-ANS on FH	3.90 ± 3.19 2.64, 5.16	2.96 ± 3.62 1.53, 4.40	-0.94 ± 3.96 -2.51, 0.63	0.66 ± 0.51 0.46, 0.87	-1.60 ± 4.04 -3.20, 0.00	0.049*
Ba-PNS	41.25 ± 4.21 39.58, 42.91	41.35 ± 4.39 39.62, 43.09	0.11 ± 3.25 -1.18, 1.39	1.47 ± 0.93 1.10, 1.83	-1.36 ± 2.98 -2.54, -0.18	0.026*
Maxilla vertical (mm)						
N-ANS	46.04 ± 3.13 44.80, 47.28	49.36 ± 3.14 48.11, 50.60	3.32 ± 3.23 2.04, 4.59	2.84 ± 1.62 2.21, 3.48	0.47 ± 2.66 -0.58, 1.52	0.366^NS^
N-PNS	65.90 ± 3.67 64.45, 67.35	70.07 ± 4.89 68.13, 72.00	4.17 ± 4.05 2.57, 5.77	3.15 ± 1.85 2.42, 3.88	1.02 ± 3.32 -0.29, 2.33	0.122^NS^
Mandible sagittal (mm)						
N-Pog on FH	-4.40 ± 9.16 -8.02, -0.78	-5.19 ± 9.43 -8.92, -1.47	-0.80 ± 4.80 -2.69, 1.10	2.66 ± 1.79 1.95, 3.36	-3.45 ± 5.22 -5.52, -1.39	0.002**
Co^(dorsal)−^PTV	31.60 ± 2.58 30.58, 32.63	32.81 ± 3.07 31.60, 34.03	1.21 ± 2.16 0.36, 2.07	1.23 ± 0.74 0.94, 1.53	-0.02 ± 1.96 -0.80, 0.75	0.951^NS^
Mandible vertical (mm)						
S-Co^(superior)^	19.53 ± 3.65 18.08, 20.97	21.08 ± 3.50 19.70, 22.47	1.56 ± 2.11 0.72, 2.39	1.42 ± 0.83 1.09, 1.75	0.14 ± 1.93 -0.62, 0.90	0.710^NS^
S-Go	68.26 ± 6.70 65.61, 70.91	74.50 ± 7.39 71.57, 77.42	6.24 ± 4.70 4.38, 8.09	5.65 ± 3.08 4.43, 6.87	0.59 ± 3.44 -0.78, 1.95	0.385^NS^
N-Me	108.70 ± 8.02 105.53, 111.87	117.00 ± 9.12 113.39, 120.60	8.30 ± 6.02 5.92, 10.68	6.38 ± 3.39 5.05, 7.72	1.91 ± 4.33 0.20, 3.63	0.030*
Dental horizontal (mm)						
U1^(incisal)^-PTV	52.22 ± 4.50 50.44, 54.01	51.14 ± 4.41 49.40, 52.89	-1.08 ± 3.96 -2.65, 0.48	3.62 ± 2.49 2.64, 4.61	-4.71 ± 4.51 -6.49, -2.92	< 0.001***
L1^(incisal)^-PTV	47.53 ± 4.15 45.89, 49.17	47.47 ± 4.46 45.71, 49.23	-0.06 ± 3.73 -1.53, 1.42	3.62 ± 2.49 2.64, 4.61	-3.68 ± 3.89 -5.22, -2.14	< 0.001***
U6^(dorsal)^-PTV	13.17 ± 3.56 11.77, 14.58	17.34 ± 3.65 15.89, 18.78	4.16 ± 2.97 2.99, 5.34	3.62 ± 2.49 2.64, 4.61	0.54 ± 3.18 -0.72, 1.80	0.386^NS^
L6^(dorsal)^-PTV	12.31 ± 3.99 10.73, 13.89	16.85 ± 3.74 15.37, 18.33	4.54 ± 3.60 3.12, 5.97	3.62 ± 2.49 2.64, 4.61	0.92 ± 3.60 -0.51, 2.34	0.197^NS^
Overjet	4.25 ± 2.75 3.16, 5.34	2.79 ± 1.29 2.28, 3.31	-1.46 ± 2.63 -2.50, -0.41	-0.31 ± 0.21 -0.40, -0.23	-1.14 ± 2.68 -2.20, -0.08	0.036*
Overbite	1.33 ± 2.03 0.52, 2.13	1.57 ± 1.44 1.00, 2.14	0.24 ± 2.21 -0.63, 1.12	0.06 ± 0.47 -0.13, 0.25	0.18 ± 2.04 -0.62, 0.99	0.643^NS^

Means (M) and standard deviations (SD) in cephalometric measurements at T1 and T2;

Net outcome/therapeutic effect, CI confidence interval, LB lower bound, UB upper bound, NS not significant

*p < 0.05; **p < 0.01; ***p < 0.001

Calculation of ΔT2–T1: positive value = increase and negative value = decrease

**Table 3:** Side effects on occlusal plane, gonial angle and lower incisor inclination in all patients

Extraction – all patients						
Measurement	T1 (M ± SD) 95% CI (LB, UB)	T2 (M ± SD) 95% CI (LB, UB)	∆T2-T1 (M ± SD) 95% CI (LB, UB)	Control (M ± SD) 95% CI (LB, UB)	Net (M ± SD) 95% CI (LB, UB)	Net *p*-value (intra)
Mandible diagonal (mm)						
Co^(dorsal)^-Pog	100.98 ± 6.98 98.22, 103.74	107.44 ± 7.00 104.67, 110.20	6.46 ± 5.53 4.27, 8.64	7.04 ± 3.78 5.55, 8.54	-0.58 ± 3.95 -2.15, 0.98	0.449^NS^
Co^(superior)^-Gn	103.80 ± 7.49 100.84, 106.76	110.74 ± 7.13 107.92, 113.56	6.94 ± 5.97 4.58, 9.30	7.29 ± 4.13 5.65, 8.92	-0.35 ± 4.08 -1.96, 1.27	0.662^NS^
Mandible angular (°)						
Ar-Go-Me	128.47 ± 5.70 126.21, 130.73	127.45 ± 6.12 125.03, 129.87	-1.02 ± 3.89 -2.56, 0.52	-1.13 ± 0.66 -1.39, -0.87	0.12 ± 3.97 -1.45, 1.68	0.881^NS^
Co^(dorsal)^-Go-Pog	122.63 ± 6.01 120.25, 125.01	121.63 ± 6.63 119.01, 124.26	-1.00 ± 3.30 -2.30, 0.31	-1.14 ± 0.66 -1.39, -0.88	0.14 ± 3.25 -1.15, 1.42	0.826^NS^
Cant of occlusal plane (°)						
SN/OP	18.19 ± 3.71 16.72, 19.65	17.74 ± 4.66 15.89, 19.58	-0.45 ± 4.01 -2.04, 1.14	-2.28 ± 2.14 -3.13, -1.43	1.83 ± 4.05 0.22, 3.43	0.027*
Dental angular (°)						
U1 / SN	103.71 ± 6.58 101.11, 106.32	97.32 ± 7.21 94.47, 100.18	-6.39 ± 7.34 -9.30, -3.49	-0.15 ± 0.85 -0.48, 0.19	-6.24 ± 7.18 -9.08, -3.40	< 0.001***
U1 / PP	108.42 ± 5.87 106.10, 110.74	103.40 ± 7.19 100.56, 106.24	-5.02 ± 7.35 -7.93, -2.11	0.05 ± 24.97 -9.82, 9.93	-5.07 ± 26.73 -15.64, 5.50	0.333^NS^
L1 / MP	91.93 ± 7.22 89.07, 94.78	89.53 ± 7.07 86.74, 92.33	-2.39 ± 7.70 -5.44, 0.65	0.01 ± 1.03 -0.40, 0.41	-2.40 ± 7.41 -5.33, 0.53	0.105^NS^
U1/ L1 (interincisal angle)	127.20 ± 10.55 123.03, 131.37	135.75 ± 8.68 132.32, 139.18	8.55 ± 11.64 3.95, 13.16	1.42 ± 1.87 0.69, 2.16	7.13 ± 10.77 2.87, 11.39	0.002**
U6 / SN	103.71 ± 6.58 101.11, 106.32	97.32 ± 7.21 94.47, 100.18	-6.39 ± 7.34 -9.30, -3.49	-0.15 ± 0.85 -0.48, 0.19	-6.24 ± 7.18 -9.08, -3.40	< 0.001***
L6 / MP	91.93 ± 7.22 89.07, 94.78	89.53 ± 7.07 86.74, 92.33	-2.39 ± 7.70 -5.44, 0.65	0.01 ± 1.03 -0.40, 0.41	-2.40 ± 7.41 -5.33, 0.53	0.105^NS^

Means (M) and standard deviations (SD) in cephalometric measurements at T1 and T2;

Net outcome/therapeutic effect, CI confidence interval, LB lower bound, UB upper bound, NS not significant

*p < 0.05; **p < 0.01; ***p < 0.001

Calculation of ΔT2–T1: positive value = increase and negative value = decrease

**Table 4:** Skeletal and dental effects in hypodivergent patients

Extraction – hypodivergent patients							
Measurement	T1 (M ± SD) 95% CI (LB, UB)	T2 (M ± SD) 95% CI (LB, UB)	∆T2-T1 (M ± SD) 95% CI (LB, UB)	Control (M ± SD) 95% CI (LB, UB)	Net (M ± SD) 95% CI (LB, UB)	Net *p*-value (intra)	Net *p*-value (inter)
Maxilla sagittal (mm)							
N-ANS on FH	4.76 ± 3.21 2.07, 7.44	4.46 ± 4,39 0.80, 8.13	-0.29 ± 3,68 -3.37, 2.78	0.63 ± 0.33 0.35, 0.90	-0.92 ± 3.65 -3.97, 2.13	0.120^NS^	0.579^NS^
Ba-PNS	42.44 ± 3.65 39.39, 45.50	44.22 ± 5.30 39.79, 48.65	1.78 ± 4.08 -1.64, 5.19	1.31 ± 0.96 0.51, 2.11	0.47 ± 3.69 -2.62, 3.56	0.097^NS^	0.036^*^
Maxilla vertical (mm)							
N-ANS	46.22 ± 3.37 43.40, 49.03	47.90 ± 4.22 44.38, 51.43	1.69 ± 3.14 -0.94, 4.31	2.55 ± 1.72 1.11, 3.99	-0.86 ± 3.50 -3.79, 2.06	0.109^NS^	0.091^NS^
N-PNS	66.53 ± 4.91 62.43, 70.64	69.34 ± 7.41 63.14, 75.53	2.80 ± 4.29 -0.79, 6.39	2.89 ± 1.90 1.30, 4.47	-0.09 ± 4.31 -3.69, 3.52	0.007**	0.269^NS^
Mandible sagittal (mm)							
N-Pog on FH	-4.73 ± 7.05 -10.62, 1.17	-3.12 ± 5.90 -8.06, 1.82	1.61 ± 4.21 -1.91, 5.13	2.78 ± 1.50 1.52, 4.03	-1.17 ± 4.73 -5.13, 2.79	0.507^NS^	0.143^NS^
Co^(dorsal)−^PTV	34.26 ± 1.42 33.07, 35.45	35.50 ± 1.50 34.25, 36.76	1.25 ± 2.21 -0.60, 3.10	1.02 ± 0.74 0.39, 1.64	0.23 ± 2.12 -1.54, 2.00	0.646^NS^	0.671^NS^
Mandible vertical (mm)							
S-Co^(superior)^	21.69 ± 3.30 18.94, 24.45	23.55 ± 3.76 20.40, 26.70	1.86 ± 2.67 -0.38, 4.09	1.24 ± 0.86 0.52, 1.96	0.62 ± 2.41 -1.39, 2.63	0.038*	0.413^NS^
S-Go	70.92 ± 6.83 65.21, 76.62	79.05 ± 10.34 70.41, 87.69	8.13 ± 6.10 3.04, 13.23	5.28 ± 2.97 2.80, 7.76	2.85 ± 4.41 -0.84, 6.54	0.003**	0.085^NS^
N-Me	105.20 ± 10.42 96.49, 113.92	113.18 ± 14.79 100.82, 125.55	7.98 ± 6.58 2.48, 13.49	5.78 ± 3.52 2.83, 8.72	2.20 ± 5.28 -2.21, 6.62	< 0.001***	0.827^NS^
Dental horizontal (mm)							
U1^(incisal)^-PTV	53.16 ± 5.09 48.91, 57.41	53.87 ± 4.69 49.95, 57.80	0.71 ± 4.45 -3.00, 4.43	3.70 ± 2.08 1.96, 5.44	-2.99 ± 5.20 -7.34, 1.36	0.148^NS^	0.205^NS^
L1^(incisal)^-PTV	47.81 ± 4.00 44.47, 51.16	49.61 ± 5.58 44.94, 54.28	1.80 ± 3.44 -1.08, 4.68	3.70 ± 2.08 1.96, 5.44	-1.90 ± 3.78 -5.06, 1.25	0.197^NS^	0.125^NS^
U6^(dorsal)^-PTV	13.04 ± 4.08 9.63, 16.46	17.98 ± 5.17 13.66, 22.30	4.94 ± 3.36 2.12, 7.75	3.70 ± 2.08 1.96, 5.44	1.23 ± 3.55 -1.73, 4.20	0.359^NS^	0.474^NS^
L6^(dorsal)^-PTV	12.05 ± 4.37 8.39, 15.70	17.25 ± 5.55 12.61, 21.89	5.21 ± 4.17 1.72, 8.69	3.70 ± 2.08 1.96, 5.44	1.50 ± 3.85 -1.71, 4.72	0.359^NS^	0.595^NS^
Overjet	4.92 ± 2.65 2.70, 7.13	3.23 ± 1.10 2.31, 4.15	-1.69 ± 2.98 -4.18, 0.81	-0.24 ± 0.22 -0.43, -0.06	-1.44 ± 3.01 -3.96, 1.08	0.218^NS^	0.714^NS^
Overbite	2.09 ± 2.12 0.32, 3.86	1.96 ± 2.00 0.29, 3.62	-0.13 ± 2.67 -2.36, 2.10	0.17 ± 0.61 -0.34, 0.68	-0.30 ± 2.58 -2.45, 1.86	0.753^NS^	0.436^NS^

Means (M) and standard deviations (SD) in cephalometric measurements at T1 and T2;

Net outcome/therapeutic effect, CI confidence interval, LB lower bound, UB upper bound, NS not significant

*p < 0.05; **p < 0.01; ***p < 0.001

Calculation of ΔT2–T1: positive value = increase and negative value = decrease

**Table 5:** Side effects on occlusal plane, gonial angle and lower incisor inclination in hypodivergent patients

Extraction–hypodivergent patients							
Measurement	T1 (M ± SD) 95% CI (LB, UB)	T2 (M ± SD) 95% CI (LB, UB)	∆T2-T1 (M ± SD) 95% CI (LB, UB)	Control (M ± SD) 95% CI (LB, UB)	Net (M ± SD) 95% CI (LB, UB)	Net *p*-value (intra)	Net *p*-value (inter)
Mandible diagonal (mm)							
Co^(dorsal)^-Pog	101.94 ± 7.55 95.63, 108.26	109.91 ± 9.61 101.87, 117.95	7.96 ± 6.58 2.46, 13.47	6.45 ± 3.83 3.25, 9.65	1.51 ± 4.57 -2.31, 5.33	0.380^NS^	0.072^NS^
Co^(superior)^-Gn	105.07 ± 8.33 98.11, 112.04	112.31 ± 10.52 103.51, 121.10	7.23 ± 7.86 0.66, 13.81	6.55 ± 4.30 2.95, 10.14	0.69 ± 5.46 -3.87, 5.25	0.005**	0.402^NS^
Mandible angular (°)							
Ar-Go-Me	123.39 ± 5.29 118.96, 127.81	122.25 ± 4.89 118.16, 126.34	-1.14 ± 2.95 -3.60, 1.33	-1.10 ± 0.75 -1.73, -0.47	-0.04 ± 3.00 -2.54, 2.47	0.974^NS^	0.900^NS^
Co^(dorsal)^-Go-Pog	117.04 ± 5.99 112.03, 122.05	115.91 ± 5.24 111.53, 120.30	-1.12 ± 2.51 -3.22, 0.97	-1.10 ± 0.75 -1.73, -0.47	-0.02 ± 2.57 -2.17, 2.13	0.980^NS^	0.870^NS^
Cant of occlusal plane (°)							
SN/OP	15.26 ± 2.84 12.89, 17.64	13.86 ± 5.09 9.61, 18.11	-1.40 ± 5.57 -6.06, 3.26	-3.09 ± 3.39 -5.92, -0.25	1.69 ± 5.83 -3.19, 6.56	0.440^NS^	0.930^NS^
Dental angular (°)							
U1 / SN	106.70 ± 7.06 100.80, 112.60	101.74 ± 8.05 95.01, 108.47	-4.96 ± 10.04 -13.36, 3.43	0.02 ± 0.41 -0.33, 0.36	-4.98 ± 9.70 -13.09, 3.13	0.190^NS^	0.563^NS^
U1 / PP	110.46 ± 5.56 105.81, 115.11	105.21 ± 8.20 98.36, 112.06	-5.25 ± 9.47 -13.17, 2.67	-11.07 ± 31.76 -37.63, 15.48	5.82 ± 35.18 -23.59, 35.24	0.654^NS^	0.174^NS^
L1 / MP	95.43 ± 6.84 89.71, 101.14	94.59 ± 7.49 88.32, 100.85	-0.84 ± 8.68 -8.10, 6.42	0.01 ± 1.13 -0.93, 0.95	-0.84 ± 8.27 -7.76, 6.07	0.781^NS^	0.490^NS^
U1/ L1 (interincisal angle)	127.05 ± 9.59 119.04, 135.06	134.74 ± 8.49 127.64, 141.83	7.69 ± 12.32 -2.61, 17.98	1.14 ± 2.05 -0.57, 2.85	6.55 ± 10.89 -2.56, 15.65	0.133^NS^	0.860^NS^
U6 / SN	71.27 ± 4.39 67.60, 74.95	80.47 ± 5.66 75.74, 85.21	9.20 ± 2.72 6.93, 11.47	0.02 ± 0.41 -0.33, 0.36	9.18 ± 2.55 7.05, 11.31	< 0.001***	0.115^NS^
L6 / MP	91.96 ± 8.91 84.51, 99.41	94.29 ± 6.11 89.18, 99.40	2.32 ± 5.62 -2.37, 7.02	0.01 ± 1.13 -0.93, 0.95	2.32 ± 6.04 -2.73, 7.37	0.314^NS^	0.964^NS^

Means (M) and standard deviations (SD) in cephalometric measurements at T1 and T2;

Net outcome/therapeutic effect, CI confidence interval, LB lower bound, UB upper bound, NS not significant

*p < 0.05; **p < 0.01; ***p < 0.001

Calculation of ΔT2–T1: positive value = increase and negative value = decrease

**Table 6:** Skeletal and dental effects in hyperdivergent patients

Extraction – hyperdivergent patients							
Measurement	T1 (M ± SD) 95% CI (LB, UB)	T2 (M ± SD) 95% CI (LB, UB)	∆T2-T1 (M ± SD) 95% CI (LB, UB)	Control (M ± SD) 95% CI (LB, UB)	Net (M ± SD) 95% CI (LB, UB)	Net *p*-value (intra)	Net *p*-value (inter)
Maxilla sagittal (mm)							
N-ANS on FH	3.54 ± 3.19 2.00, 5.08	2.33 ± 3.16 0.81, 3.86	-1.21 ± 4.14 -3.21, 0.79	0.68 ± 0.58 0.40, 0.96	-1.89 ± 4.25 -3.94, 0.16	0.049*	0.579^NS^
Ba-PNS	40.74 ± 4.42 38.62, 42.87	40.15 ± 3.43 38.49, 41.80	-0.60 ± 2.65 -1.88, 0.68	1.53 ± 0.93 1.08, 1.98	-2.13 ± 2.33 -3.25, -1.01	0.026*	0.036*
Maxilla vertical (mm)							
N-ANS	45.97 ± 3.12 44.46, 47.47	49.97 ± 2.45 48.79, 51.15	4.00 ± 3.09 2.51, 5.49	2.97 ± 1.60 2.20, 3.74	1.03 ± 2.08 0.03, 2.04	0.366^NS^	0.091^NS^
N-PNS	65.63 ± 3.14 64.12, 67.14	70.38 ± 3.59 68.65, 72.11	4.75 ± 3.91 2.86, 6.63	3.26 ± 1.87 2.36, 4.16	1.49 ± 2.81 0.13, 2.84	0.122^NS^	0.269^NS^
Mandible sagittal (mm)							
N-Pog on FH	-4.26 ± 10.08 -9.12, 0.60	-6.07 ± 10.59 -11.17, -0.97	-1.81 ± 4.76 -4.10, 0.49	2.61 ± 1.93 1.68, 3.54	-4.42 ± 5.23 -6.94, -1.90	0.002**	0.143^NS^
Co^(dorsal)−^PTV	30.49 ± 2.10 29.47, 31.50	31.68 ± 2.86 30.30, 33.06	1.20 ± 2.20 0.13, 2.26	1.33 ± 0.74 0.97, 1.68	-0.13 ± 1.95 -1.07, 0.81	0.951^NS^	0.671^NS^
Mandible vertical (mm)							
S-Co^(superior)^	18.62 ± 3.47 16.94, 20.29	20.04 ± 2.89 18.65, 21.44	1.43 ± 1.89 0.52, 2.34	1.49 ± 0.84 1.09, 1.89	-0.06 ± 1.73 -0.90, 0.77	0.710^NS^	0.413^NS^
S-Go	67.15 ± 6.50 64.02, 70.28	72.58 ± 4.93 70.21, 74.96	5.44 ± 3.89 3.56, 7.31	5.81 ± 3.19 4.27, 7.34	-0.37 ± 2.51 -1.58, 0.84	0.385^NS^	0.085^NS^
N-Me	110.17 ± 6.55 107.01, 113.32	118.60 ± 5.09 116.15, 121.05	8.43 ± 5.96 5.56, 11.30	6.64 ± 3.39 5.01, 8.27	1.79 ± 4.02 -0.14, 3.73	0.030*	0.827^NS^
Dental horizontal (mm)							
U1^(incisal)^-PTV	51.83 ± 4.32 49.75, 53.91	49.99 ± 3.85 48.14, 51.84	-1.84 ± 3.59 -3.57, -0.11	3.59 ± 2.69 2.29, 4.89	-5.43 ± 4.12 -7.42, -3.44	< 0.001***	0.205^NS^
L1^(incisal)^-PTV	47.41 ± 4.32 45.33, 49.49	46.57 ± 3.70 44.79, 48.35	-0.84 ± 3.65 -2.60, 0.92	3.59 ± 2.69 2.29, 4.89	-4.43 ± 3.78 -6.26, -2.61	< 0.001***	0.125^NS^
U6^(dorsal)^-PTV	13.23 ± 3.43 11.57, 14.88	17.07 ± 2.94 15.65, 18.48	3.84 ± 2.82 2.48, 5.20	3.59 ± 2.69 2.29, 4.89	0.25 ± 3.07 -1.23, 1.73	0.386^NS^	0.474^NS^
L6^(dorsal)^-PTV	12.41 ± 3.95 10.51, 14.32	16.68 ± 2.84 15.31, 18.05	4.26 ± 3.42 2.62, 5.91	3.59 ± 2.69 2.29, 4.89	0.67 ± 3.58 -1.05, 2.40	0.197^NS^	0.595 ^NS^
Overjet	3.97 ± 2.82 2.61, 5.33	2.61 ± 1.35 1.96, 3.26	-1.36 ± 2.55 -2.59, -0.13	-0.34 ± 0.20 -0.44, -0.25	-1.02 ± 2.61 -2.27, 0.24	0.036*	0.714^NS^
Overbite	1.01 ± 1.96 0.06, 1.95	1.41 ± 1.17 0.84, 1.97	0.40 ± 2.05 -0.59, 1.39	0.01 ± 0.41 -0.19, 0.21	0.39 ± 1.81 -0.48, 1.26	0.643^NS^	0.436^NS^

Means (M) and standard deviations (SD) in cephalometric measurements at T1 and T2;

Net outcome/therapeutic effect, CI confidence interval, LB lower bound, UB upper bound, NS not significant

*p < 0.05; **p < 0.01; ***p < 0.001

Calculation of ΔT2–T1: positive value = increase and negative value = decrease

**Table 7:** Side effects on occlusal plane, gonial angle and lower incisor inclination in hyperdivergent patients

Extraction – hyperdivergent patients							
Measurement	T1 (M ± SD) 95% CI (LB, UB)	T2 (M ± SD) 95% CI (LB, UB)	∆T2-T1 (M ± SD) 95% CI (LB, UB)	Control (M ± SD) 95% CI (LB, UB)	Net (M ± SD) 95% CI (LB, UB)	Net *p*-value (intra)	Net *p*-value (inter)
Mandible diagonal (mm)							
Co^(dorsal)^-Pog	100.57 ± 6.90 97.25, 103.90	106.40 ± 5.56 103.72, 109.08	5.82 ± 5.08 3.37, 8.27	7.29 ± 3.84 5.44, 9.14	-1.47 ± 3.41 -3.11, 0.18	0.449^NS^	0.072^NS^
Co^(superior)^-Gn	103.27 ± 7.28 99.76, 106.77	110.08 ± 5.38 107.49, 112.68	6.82 ± 5.23 4.30, 9.34	7.60 ± 4.14 5.61, 9.60	-0.78 ± 3.43 -2.44, 0.87	0.662^NS^	0.402^NS^
Mandible angular (°)							
Ar-Go-Me	130.61 ± 4.45 128.47, 132.75	129.64 ± 5.27 127.10, 132.18	-0.97 ± 4.30 -3.04, 1.10	-1.15 ± 0.64 -1.45, -0.84	0.18 ± 4.38 -1.93, 2.29	0.881^NS^	0.900^NS^
Co^(dorsal)^-Go-Pog	124.98 ± 4.29 122.92, 127.05	124.04 ± 5.67 121.31, 126.78	-0.94 ± 3.65 -2.70, 0.82	-1.15 ± 0.63 -1.46, -0.84	0.21 ± 3.56 -1.51, 1.92	0.826^NS^	0.870^NS^
Cant of occlusal plane (°)							
SN/OP	19.42 ± 3.36 17.80, 21.04	19.37 ± 3.43 17.71, 21.02	-0.05 ± 3.26 -1.62, 1.52	-1.94 ± 1.32 -2.57, -1.30	1.88 ± 3.23 0.33, 3.44	0.027*	0.930^NS^
Dental angular (°)							
U1 / SN	102.46 ± 6.13 99.51, 105.41	95.46 ± 6.14 92.51, 98.42	-6.99 ± 6.11 -9.94, -4.05	-0.22 ± 0.98 -0.69, 0.26	-6.78 ± 6.07 -9.70, -3.85	< 0.001***	0.563^NS^
U1 / PP	107.56 ± 5.92 104.71, 110.41	102.64 ± 6.81 99.35, 105.92	-4.92 ± 6.57 -8.09, -1.75	4.74 ± 20.73 -5.26, 14.73	-9.66 ± 21.81 -20.17, 0.86	0.333^NS^	0.174^NS^
L1 / MP	90.45 ± 7.03 87.06, 93.84	87.41 ± 5.86 84.58, 90.23	-3.05 ± 7.40 -6.61, 0.52	0.01 ± 1.02 -0.49, 0.50	-3.05 ± 7.16 -6.50, 0.40	0.105^NS^	0.490^NS^
U1/ L1 (interincisal angle)	127.26 ± 11.18 121.87, 132.65	136.18 ± 8.95 131.86, 140.49	8.92 ± 11.67 3.29, 14.54	1.54 ± 1.83 0.66, 2.43	7.37 ± 11.01 2.07, 12.68	0.002**	0.860^NS^
U6 / SN	69.41 ± 6.19 66.42, 72.39	75.09 ± 4.71 72.82, 77.36	5.68 ± 5.18 3.19, 8.18	-0.22 ± 0.98 -0.69, 0.26	5.90 ± 5.38 3.31, 8.50	< 0.001***	0.115^NS^
L6 / MP	98.27 ± 4.52 96.09, 100.45	100.49 ± 4.65 98.25, 102.73	2.22 ± 5.19 -0.28, 4.72	0.01 ± 1.02 -0.49, 0.50	2.22 ± 5.06 -0.22, 4.65	0.035*	0.964^NS^

Means (M) and standard deviations (SD) in cephalometric measurements at T1 and T2;

Net outcome/therapeutic effect, CI confidence interval, LB lower bound, UB upper bound, NS not significant

*p < 0.05; **p < 0.01; ***p < 0.001

Calculation of ΔT2–T1: positive value = increase and negative value = decrease

In hypodivergent patients, posterior (S-Go) and anterior face height (N-Me) increased almost equally by an average 2.85 and 2.20 mm, respectively. In contrast, only anterior facial height (N-Me) was significantly increased in hyperdivergent patients. In hypodivergent patients, the overbite decreased by an average of 0.30 mm, while in hyperdivergent patients there was an increase of about 0.40 mm. These figures showed an almost parallel increase in lower facial height in hypodivergent patients, while only anterior facial height increased in hyperdivergent patients.

In hypodivergent patients, upper incisors (U1/SN) insignificantly retroclined and maxillary first molars (U6^(dorsal)^-PTV) migrated mesially whereas maxillary first molars (U6/SN) showed significant mesial tipping. In hyperdivergent patients, upper incisors significantly retroclined and maxillary first molars migrated mesially. Maxillary first molars also exhibited significant mesial tipping in hypodivergent patients. No significant difference was found between hypo- and hyperdivergent patients.

In both groups, the gonial angle showed only small changes. Anterior canting of the occlusal plane occurred in both groups but was more pronounced in hyperdivergent patients.

## Discussion

Data on upper first premolar extraction during Class II camouflage treatment in hypo- and hyperdivergent patients are rare in orthodontic literature. Thus, our study adds relevant data to this topic. However, comparison with other results remains difficult because of different study designs. A power analysis revealed that 17 patients were necessary for each group. We have increased this number slightly, but the limitation remains that results still lack a certain amount of generalizability. Larger patient numbers would have been feasible but were not available, not least due to dropouts. Since the study design is easy to follow, it might be possible to acquire larger groups with an inter-center approach.

The “Net effect” used in this study was calculated by using data of a longitudinal survey of unaffected facial growth [14]. These data were obtained from a non-homogeneous sample of Caucasian individuals. These individuals were between 4 and 20 years old, but only age-matched data were used for our study. The ideal control with untreated Class II subjects followed up on a regular basis is and will be unavailable. Therefore, limitations must be acknowledged when employing data from growth studies.

The selective removal of permanent teeth to camouflage a skeletal discrepancy is one of several treatment options for the correction of Class II malocclusions [12]. It has been proven that the resulting occlusion after orthodontic camouflage is stable in patients with mandibular deficiency [23]. The resulting combination of posterior distocclusion and anterior neutral occlusion does not lead to any functional limitations [24]. Gianelly et al. also found that condyle position after camouflage treatment did not differ from that of untreated control subjects [25].

It has been reported that dolichocephalic patients may have a steepening of the occlusal plane and subsequent clockwise rotation of the mandible, resulting in an additional increase of facial height in an already long-face patient [24]. Our results showed the occurrence of anterior canting of the occlusal plane in both groups, but it was significant only in hyperdivergent patients (*p* = 0.027).

Janson et al. [26] found that Class II camouflage treatment provided better occlusal results than a four-premolar-extraction protocol, as it requires more effort to achieve neutral occlusion due to the need for additional anchorage. In any case, there is a measurable anchorage loss of the first molars after premolar extraction and residual gap closure. In the hypodivergent group, upper incisors (U1/SN) were only slightly retroclined by -4.98° and the maxillary first molars (U6^(dorsal)^-PTV) had migrated mesially by 1.23 mm, whereas maxillary first molars (U6/SN) showed significant (*p* < 0.001) mesial tipping of 9.18°. In hyperdivergent patients, upper incisors were significantly (*p* < 0.001) retroclined by -6.78° and maxillary first molars had migrated mesially by 0.25 mm. In hyperdivergent patients, maxillary first molars also showed a significant (*p* < 0.001) mesial tipping of 5.90°. Although differences existed between hypo- and hyperdivergent patients, they were not significant.

Nevertheless, the results showed that the reciprocal mechanics inevitably lead to anchorage loss of the posterior teeth during residual space closure. In their randomized controlled clinical trial (RCT), Stivaros et al. [27] investigated patients after maxillary first premolar extraction therapy. Patients were allocated to two groups depending on the anchorage, either with a transpalatal arch (TPA) or a Nance appliance. Although mesial migration of maxillary first molars was observed in both groups, the difference was not significant. Mesial migration of maxillary first molars was mean 0.98 mm in TPA-patients and 0.72 mm in Nance-patients. These figures are close to those found in this study. Feldmann and Bondemark [28] designed an RCT with maxillary first molar anchorage through a TPA. Retraction of the anterior segment after extraction of maxillary first premolars lead to mesial migration of the maxillary first molars of 2.0 mm on average, which was even more than in the hypodivergent patients of this study.

In a study of patients with upper first premolar extraction, Liu et al. [29] observed anchorage loss of first molars during space closure with sliding mechanics of 1.65 mm on average, although a TPA was present. In a retrospective cephalometric study of patients with upper first premolar extraction, Zablocki et al. [30] investigated a group with a welded TPA as anchorage during retraction of the anterior segment versus a control group without additional anchorage. They described anchorage loss of the maxillary first molars of 4.1 mm and 4.5 mm, respectively, thus always greater than in our patients where the anchorage loss always appeared relatively small. This difference may due to different study designs or to the treatment method with anchorage provided by the asymmetric "T" archwire [18] with dedicated bends.

The present results show an almost parallel increase in lower face height in hypodivergent patients, while only anterior facial height increased in hyperdivergent patients. This contradicts the idea that extraction of the upper premolars in hyperdivergent types is suitable to "close the bite" [13]. Luecke and Johnston [31] investigated the effects of maxillary first premolar extraction and incisor retraction using an edgewise technique on the mandibular position in 42 patients. In contrast to our results, they described that the mandible rotated anteriorly in 70 per cent of the subjects.

In our hypodivergent patients, the gonial angle and “modified" gonial angle (Co^(dorsal)^-Go-Pog) showed a very small net decrease while hyperdivergent patients experienced a slight increase of < 0.5°. Meral et al. [32] also found a decrease of the gonial angle by 0.5°. However, these authors did not differentiate between hypo- and hyperdivergent patients. Another investigation [24] reported a slight increase of the gonial angle by 0.13°.

## Conclusions

- Camouflage treatment did not prevent bite opening in hyperdivergent patients but does not close the bite in hypodivergent patients.
- Camouflage treatment has no measurable effect on occlusal plane and gonial angle in hypo- and hyperdivergent patients.
- The facial growth type has no influence on the occurrence of anchorage loss during retraction of incisors and canines during camouflage treatment.

## Data Availability

Not applicable.
